# Immunodominant linear B cell epitopes in the spike and membrane proteins of SARS-CoV-2 identified by immunoinformatics prediction and immunoassay

**DOI:** 10.1038/s41598-021-99642-w

**Published:** 2021-10-14

**Authors:** Kanokporn Polyiam, Waranyoo Phoolcharoen, Namphueng Butkhot, Chanya Srisaowakarn, Arunee Thitithanyanont, Prasert Auewarakul, Tawatchai Hoonsuwan, Marasri Ruengjitchatchawalya, Phenjun Mekvichitsaeng, Yaowaluck Maprang Roshorm

**Affiliations:** 1 Division of Biotechnology, School of Bioresources and Technology, King Mongkut’s University of Technology Thonburi, Bangkok, Thailand; 2 Research Unit for Plant-Produced Pharmaceuticals and Department of Pharmacognosy and Pharmaceutical Botany, Faculty of Pharmaceutical Sciences, Chulalongkorn University, Bangkok, Thailand; 3 Department of Microbiology, Faculty of Science, Mahidol University, Bangkok, Thailand; 4 Department of Microbiology, Faculty of Medicine Siriraj Hospital, Mahidol University, Bangkok, Thailand; 5 B.F. Feed Company Limited, Prachachuen Road, Thung Song Hong, Lak Si, Bangkok, Thailand; 6 Bioinformatics and Systems Biology Program, School of Bioresources and Technology, King Mongkut’s University of Technology Thonburi, Bangkok, Thailand; 7 Pilot Plant Development and Training Institute, King Mongkut’s University of Technology Thonburi, Bangkok, Thailand

**Keywords:** Computational biology and bioinformatics, Immunology, Microbiology

## Abstract

SARS-CoV-2 continues to infect an ever-expanding number of people, resulting in an increase in the number of deaths globally. With the emergence of new variants, there is a corresponding decrease in the currently available vaccine efficacy, highlighting the need for greater insights into the viral epitope profile for both vaccine design and assessment. In this study, three immunodominant linear B cell epitopes in the SARS-CoV-2 spike receptor-binding domain (RBD) were identified by immunoinformatics prediction, and confirmed by ELISA with sera from *Macaca fascicularis* vaccinated with a SARS-CoV-2 RBD subunit vaccine. Further immunoinformatics analyses of these three epitopes gave rise to a method of linear B cell epitope prediction and selection. B cell epitopes in the spike (S), membrane (M), and envelope (E) proteins were subsequently predicted and confirmed using convalescent sera from COVID-19 infected patients. Immunodominant epitopes were identified in three regions of the S2 domain, one region at the S1/S2 cleavage site and one region at the C-terminus of the M protein. Epitope mapping revealed that most of the amino acid changes found in variants of concern are located within B cell epitopes in the NTD, RBD, and S1/S2 cleavage site. This work provides insights into B cell epitopes of SARS-CoV-2 as well as immunoinformatics methods for B cell epitope prediction, which will improve and enhance SARS-CoV-2 vaccine development against emergent variants.

## Introduction

Coronavirus disease 2019 (COVID-19) is a novel disease, discovered during its initial outbreak in December 2019 in Wuhan, China^1^. Subsequently, severe acute respiratory syndrome coronavirus 2 (SARS-CoV-2) was found to be the causative agent of COVID-19^3^. It is a contagious and devastating disease that can be transmitted from human to human and was later announced as a pandemic disease by WHO^2^. The virus continues to spread and cause health issues worldwide. Vaccination is an effective means to prevent the infection and limit the spread of the virus.

SARS-CoV-2 virus is a member of the *Betacoronavirus* genus. The genus contains two other coronaviruses, SARS-CoV and MERS-CoV, which can infect humans^4^. It is a positive-, single-stranded RNA virus that is characterized by a spherical shape^5^. To date, new emerging variants of SARS-CoV-2 have been reported and four have been classified as variants of concern, which include B.1.1.7 (Alpha), B.1.351 (Beta), P.1 (Gamma), and B.1.617.2 (Delta)^6^. These variants are circulating in numerous countries and some of which are associated with increased transmissibility^7–9^. Importantly, these variants may affect vaccine efficacy. It has been demonstrated that these emerging variants confer increased resistance to the antibodies generated through a previous natural infection and vaccination^7,8,10^.

The SARS-CoV-2 particle consists of multiple structural proteins; however, spike (S), membrane (M), envelope (E), and nucleocapsid (N) proteins are prevalent^11^. Among these, the S protein has been the main target for vaccine development. It is composed of 1273 amino acid residues and divided into two subunits: S1 and S2^12^. The S1 subunit consists of the N-terminal domain (NTD), receptor-binding domain (RDB), and C-terminal domain (CTD)^12^. The RBD spans from amino acid residue 319 to amino acid residue 541 on the S protein and it is responsible for interacting with the host cell receptor, Angiotensin-converting enzyme 2 (ACE2), to facilitate viral entry to the cell^12,13^. The S1 subunit, particularly RBD, is thus considered the most important target for vaccine development. Indeed, neutralizing antibodies against the S protein have been characterized from individuals infected with SARS-CoV-2 ^14–16^. Other main structural proteins embedded on the coronavirus membrane, M and E proteins, also play important roles in the viral assembly, budding, and replication of virus particles^17,18^. Antibodies against these two proteins have also been detected in COVID-19 patients^16^; however, it is not yet clear whether these antibodies confer neutralizing capacity. Therefore, they have not been the focus of vaccination strategies.

Generation of neutralizing antibody responses, which can neutralize the virus and thus prevent virus infection, are reliant on immunogenic B cell epitopes within the S protein^19^. Identification of these immunogenic epitopes within a target protein can better inform and guide vaccine design. B cell epitopes can be divided into two forms: (i) a linear epitope, which is a continuous amino acid sequence fragment of an antigen, (ii) a conformational epitope, by which the residues crucial for antibody recognition are in close proximity within the folded protein 3-D structure^20^. Most of the B cell epitopes are identified using experimental methods, by which either overlapping peptides or short peptides are used in the immunoassays. However, this approach is labor-intensive, and is both costly and time-consuming.

Immunoinformatics is an alternative and powerful tool for the identification of potential B and T cell epitopes. Currently, many tools and methods are available for the prediction of linear and conformational B cell epitopes. Among the recommended tools and resources for epitope prediction, the IEDB database provides multiple tools for the prediction of linear B cell epitopes and other associated parameters of B cell epitopes^21^. Several investigations have reported putative B cell epitopes of the SARS-CoV-2 proteins predicted by immunoinformatics approach^22–25^. Although immunoinformatics tools can be used to explore potential epitopes, the peptides predicted by these methods cannot be claimed as epitopes and further experimental validation is still required to confirm whether they are genuine epitopes. Nevertheless, employing immunoinformatics for epitope prediction and screening could reduce the number of peptides used in experimental assays, thus saving both time and cost.

In this work, we aimed to identify linear B cell epitopes in the 3 main structural proteins of SARS-CoV-2 by coupling the immunoinformatics method for epitope prediction with immunoassays. B cell epitopes in the RBD region predicted by the BepiPred-2.0 tool were tested with the sera from cynomolgus macaques *(M. fascicularis*) vaccinated with a plant-produced SARS-CoV-2 RBD subunit vaccine. Competitive inhibition of RBD-ACE2 binding was performed to investigate neutralizing potentials of antibodies targeting the RBD immunodominant epitopes. Next, other characteristics of the RBD immunodominant epitopes were analyzed using multiple immunoinformatics tools. These results were then used to develop a novel immunoinformatics method for B cell epitope prediction and selection. Potential B cell epitopes in the proteins S, M, and E were predicted. The predicted B cell epitopes were verified experimentally using convalescent sera from COVID-19 patients. Finally, we then applied this workflow to identify epitopes in the S proteins of SARS-CoV-2 variants of concern (B.1.1.7, B.1.351, P.1. and B.1.617.2). Here, we demonstrate that immunoinformatics is a potent tool for a rapid and precise identification of B cell epitopes from the SARS-CoV-2 proteins, suggesting its future utility with other pathogens.

## Results

### Three immunodominant linear B cell epitopes in the SARS-CoV-2 RBD recognized by antibodies from vaccinated macaques

To identify linear B cell epitopes in the RBD of SARS-CoV-2 S protein, we employed an immunoinformatics tool. Using the BepiPred-2.0 server revealed 8 potential linear B cell epitopes within the RBD (Table 1). Two pairs of epitopes were found to be adjacent to each other, and were combined into one long sequence, giving rise to two more epitopes, CoV2_S-11 and CoV2_S-12. The peptides were then tested by ELISA for their ability to bind antibodies using sera samples from 8 *Cynomolgus macaques* vaccinated with plant-produced RBD subunit vaccine and 5 sera from monkeys in the control group ^26^.

**Table 1:** Profiles of the B cell epitopes in the S, M and E proteins of the SARS-CoV-2 identified by immunoinformatics approach and validated with COVID-19 convalescent sera.

Epitope/peptide name	Start–End position	Sequence	Length	Prediction method	Epitope-responding sera**
CoV2_S-1	16–52	VNLTTRTQLPPAYTNSFTRGVYYPDKVFRSSVLHSTQ	37	Combined	6/20
CoV2_S-1.1	16–33	VNLTTRTQLPPAYTNSFT	18	A	2/20
CoV2_S-1.2	36–44	VYYPDKVFR	9	C	5/20
CoV2_S-1.3	45–52	SSVLHSTQ	8	B	3/20
CoV2_S-2	59–81	FSNVTWFHAIHVSGTNGTKRFDN	23	A	6/20
CoV2_S-3	141–163	LGVYYHKNNKSWMESEFRVYSSA	23	A	8/20
CoV2_S-4	180–190	EGKQGNFKNLR	11	A	3/20
CoV2_S-5	206–222	KHTPINLVRDLPQGFSA	17	C	6/20
CoV2_S-6	248–260	YLTPGDSSSGWTA	13	A	4/20
CoV2_S-7	292–302	ALDPLSETKCT	11	B	7/20
**CoV2_S-8**	**331–356**	**NITNLCPFGEVFNATRFASVYAWNRK**	**26**	**–**	**7/20**
**CoV2_S-9**	**370–395**	**NSASFSTFKCYGVSPTKLNDLCFTNV**	**26**	**B**	**6/20**
**CoV2_S-10**	**404–424**	**GDEVRQIAPGQTGKIADYNYK**	**21**	**A**	**2/20**
**CoV2_S-11**	**439–478**	**NNLDSKVGGNYNYLYRLFRKSNLKPFERDISTEIYQAGST**	**41**	**Combined**	**3/20**
**CoV2_S-11.1**	**439–451**	**NNLDSKVGGNYNY**	**13**	**–**	**2/20**
**CoV2_S-11.2**	**455–478**	**LFRKSNLKPFERDISTEIYQAGST**	**24**	**A**	**6/20**
**CoV2_S-12**	**483–507**	**VEGFNCYFPLQSYGFQPTNGVGYQP**	**25**	**Combined**	**4/20**
**CoV2_S-12.1**	**483–493**	**VEGFNCYFPLQ**	**11**	**–**	**3/20**
**CoV2_S-12.2**	**496–507**	**GFQPTNGVGYQP**	**12**	**B**	**6/20**
**CoV2_S-13**	**516–535**	**ELLHAPATVCGPKKSTNLVK**	**20**	**A**	**4/20**
CoV2_S-14	614–622	DVNCTEVPV	9	B	4/20
CoV2_S-15	673–709	SYQTQTNSPRRARSVASQSIIAYTMSLGAENSVAYSN	37	Combined	9/20
CoV2_S-15.1	673–691	SYQTQTNSPRRARSVASQS	18	A	9/20
CoV2_S-15.2	694–709	AYTMSLGAENSVAYSN	16	A	4/20
CoV2_S-16	731–750	MTKTSVDCTMYICGDSTECS	20	Combined	1/20
CoV2_S-16.1	731–738	MTKTSVDC	8	B	6/20
CoV2_S-16.2	743–750	CGDSTECS	8	B	5/20
CoV2_S-17	786–815	KQIYKTPPIKDFGGFNFSQILPDPSKPSKR	30	Combined	14/20
CoV2_S-17.1	786–800	KQIYKTPPIKDFGGF	15	C	5/20
CoV2_S-17.2	806–815	LPDPSKPSKR	10	A	4/20
CoV2_S-18	1035–1051	GQSKRVDFCGKGYHLMSFPQSAPHG	25	Combined	3/20
CoV2_S-18.1	1035–1038	GQSKRVDFCGKG	12	B	4/20
CoV2_S-18.2	1045–1051	PQSAPHG	7	B	6/20
CoV2_S-19	1114–1128	IITTDNTFVSGNCDV	15	B	4/20
CoV2_S-20	1133–1172	VNNTVYDPLQPELDSFKEELDKYFKNHTSPDVDLGDISGI	40	A	14/20
CoV2_S-21	1238–1270	TSCCSCLKGCCSCGSCCKFDEDDSEPVLKGVKL	33	Combined	18/20
CoV2_S-21.1	1238–1255	TSCCSCLKGCCSCGSCCK	18	B	18/20
CoV2_S-21.2	1252–1270	SCCKFDEDDSEPVLKGVKL	19	A	7/20
CoV2_M-1	5–20	NGTITVEELKKLLEQW	16	A	2/20
CoV2_M-2	198–208	RYRIGNYKLNTDHSSSSDNIA	21	A	13/20
CoV2_E-1	53–75	KPSFYVYSRVKNLNSSRVPDLLV	23	A	5/16
CoV2_E-1.1	57–71	YVYSRVKNLNSSRVP	15	A	0/16

Epitopes in the RBD are highlighted in bold.

**Convalescent serum with the OD450 higher than OD450 mean of healthy control group + SD.

**Figure 1:**
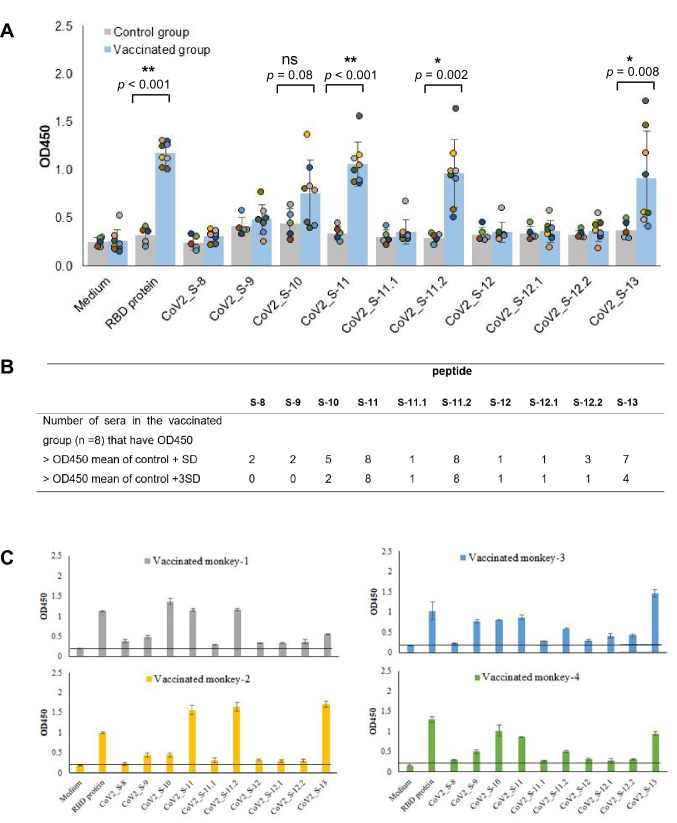
Antibody responses against predicted RBD epitopes in sera from vaccinated monkeys. (**A**) Antibody response against each predicted B cell epitope in the RBD. (**B**) Number of sera in the vaccinated group reacting with each peptide based on the criteria set. (**C**) Patterns of antibody responses in different monkeys. ELISA was performed with the synthetic peptide of each predicted epitope to determine antibody response in eight monkeys vaccinated with plant-produced RBD subunit and five control monkeys. Sera were diluted 1:100. * and ** indicates OD450 values in the control and vaccinated groups that are significantly different with *p*-value < 0.05 and 0.001, respectively. ns: not statistically significant.

Sera from vaccinated macaques showed reactivity with the complete RBD protein and a distinct group of peptides (Fig. 1A). Statistical analysis revealed that antibody responses against epitopes CoV2_S-11, CoV2_S-11.2 and CoV2_S-13 were significantly different between the vaccinated and control groups (*p* < 0.01). To further define a positive antibody response for an individual peptide, the OD450 obtained from a single vaccinated animal’s sera was compared to that of (i) OD450 mean of control group + SD, and (ii) OD450 mean of control group + 3SD (Fig. 1B). Peptides CoV2_S-11 and CoV2_S-11.2 showed reactivity with all 8 sera of the vaccinated group using both criteria. Peptide CoV2_S-13 showed reactivity with only 7 and 4 serum samples of the vaccinated group according to the criteria (i) and (ii), respectively. Although the antibody response against epitope CoV2_S-10 in the vaccinated group was not significantly different from that of the control group (*p* = 0.08), 5 and 2 sera from vaccinated monkeys showed reactivity with peptide CoV2_S-10 based on the analyses with criteria (i) and (ii), respectively.

Together, these results showed that two epitopes, CoV2_S-11 and CoV2_S-11.2, were recognized as the most immunodominant epitopes in the RBD subunit, followed by CoV2_S-13 and CoV2_S-10, respectively. It is noteworthy that epitope CoV2_S-11.2 is part of epitope CoV2_S-11, suggesting that epitope CoV2_S-11.2 is the core epitope of epitope CoV2_S-11. However, most of the monkey sera exhibited a slightly higher OD450 with peptide CoV2_S-11 than with peptide CoV2_S-11.2. Additionally, we observed that the patterns of antibody response against immunodominant epitopes in different macaques were different (Fig. 1C).

### Depiction of the immunodominant epitopes in the SARS-CoV-2 S protein

**Figure 2:**
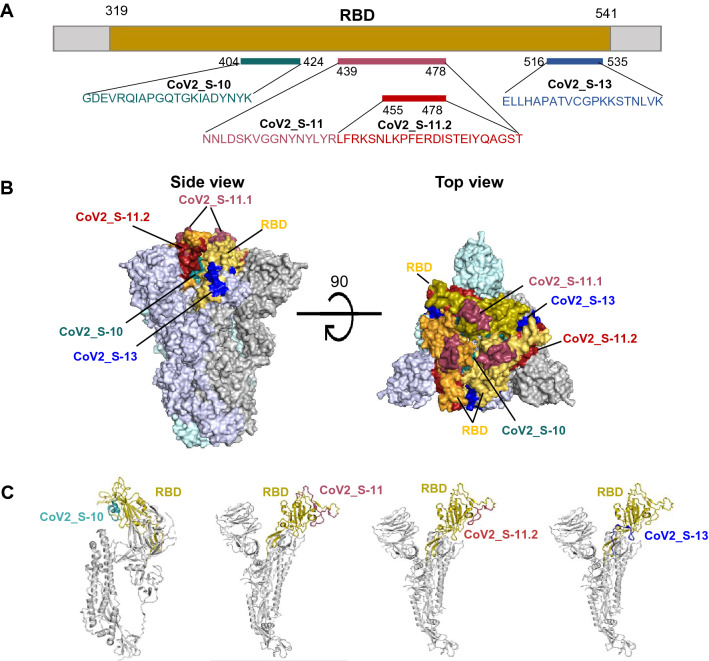
Sequences and locations of the immunodominant B cell epitopes in the RBD. (**A**) Sequences and positions of the three immunodominant epitopes in the RBD. (**B**) Surface representation of the immunodominant epitopes on the SARS-CoV-2 S trimer**. **(**C**) Locations of the immunodominant epitopes of the SARS-CoV-2 S monomer. The epitopes are labeled on the closed conformation of the 3-D S protein of SARS CoV-2 (PDB 6ZB5) using PyMOL 2.3.4 program. The RBD and immunodominant B cell epitopes are colored differentially as shown.

The positions and sequences of the three immunodominant epitopes in the RBD are shown in Fig. 2A. Locations and surface representation of the epitopes were depicted on the 3-dimensional structure of the trimeric SARS-CoV-2 S protein in the closed conformation (PDB, identifier 6ZB5^27^). The majority of the CoV2_S-11 and CoV2_S-13 epitopes’ residues are exposed on the surface of the S protein. For epitope CoV2_S-10, only a few residues are exposed on the surface (Fig. 2B). In addition, labeling the epitopes on the 3-D structure of monomeric S protein demonstrates that the three immunodominant epitopes are composed either partly or entirely of coil structure (Fig. 2C).

### Competitive inhibition of RBD-ACE2 binding by RBD-specific antibodies and peptides

We further investigated whether the antibodies targeting these RBD immunodominant epitopes could inhibit the interaction between the RBD and ACE2, by competitive ELISA. Plates were coated with human ACE2 protein and then tested with the RBD protein prepared in 3 different conditions: (i) RBD alone, (ii) RBD mixed with monkey serum (diluted 1:500), and (iii) RBD mixed with monkey serum and peptide (Fig. 3A). The RBD produced by HEK 293 cells could efficiently bind to ACE2 in a dose-dependent manner (Supplementary Fig. 1). Sera from the vaccinated monkeys (number 1, 2 and 4) were used in the assay and their antibody responses against individual immunodominant epitope are shown (Fig. 3B, left panel). All three sera tested could inhibit the interaction between the RBD and ACE2 as shown by the decrease of OD450 in the condition containing RBD and serum (Fig. 3B, right panel).

**﻿Figure 3:**
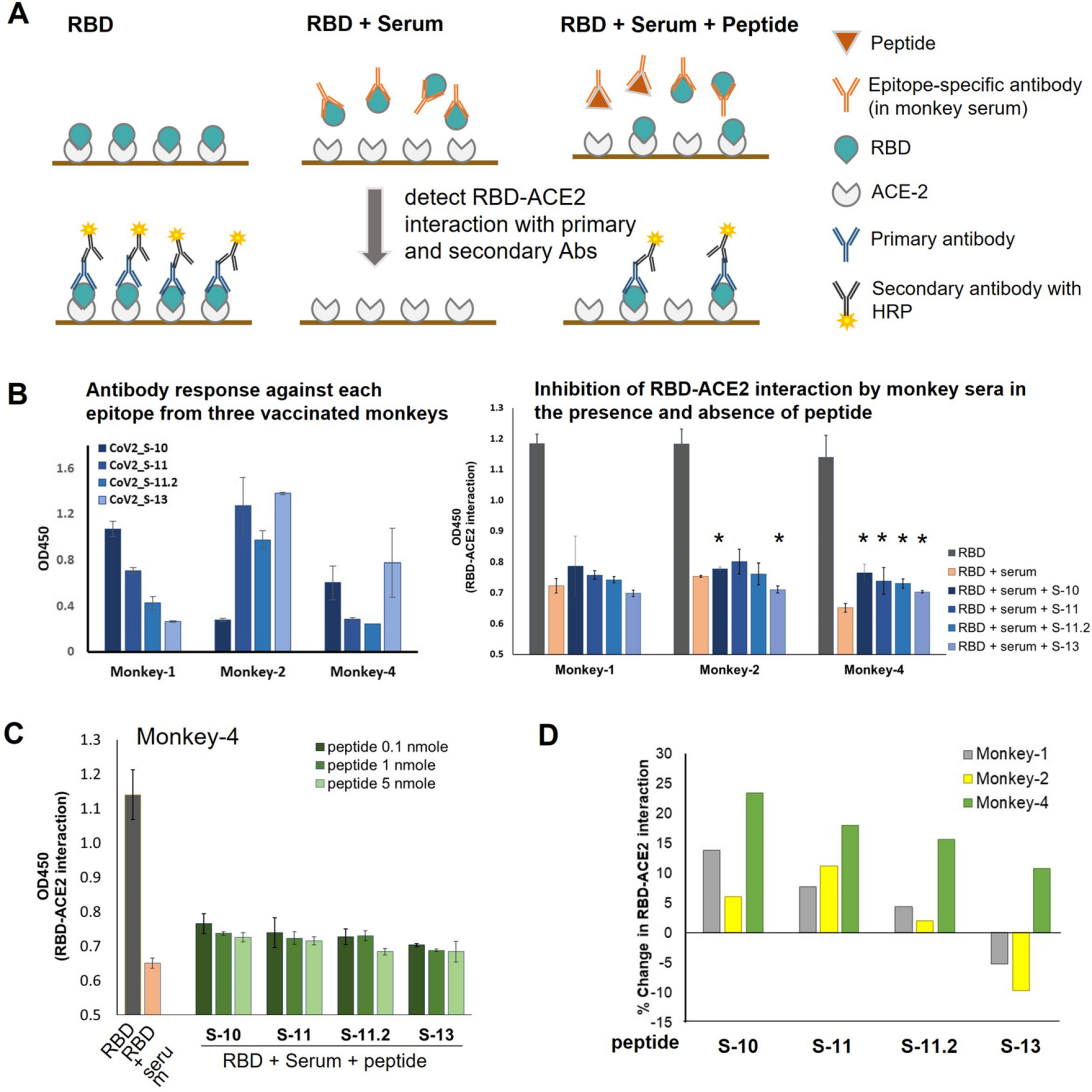
Competitive inhibition of RBD-ACE2 binding. (**A**) Schematic representation of the competitive inhibition of RBD-ACE2 interaction. The assay was performed based on the ELISA method. ELISA plates were coated with human ACE2. Binding between the RBD and ACE2 was measured in three different conditions: (i) RBD alone, (ii) RBD with monkey serum (diluted 1:500), and (iii) RBD with monkey serum and peptide. (**B**) Antibody response against each epitope observed in the three monkey sera used in the assay (left panel) and inhibition of RBD-ACE2 interaction by monkey antibodies in the presence and absence of peptide (right panel). * indicates a significant difference between the conditions with and without peptide (*p* < 0.05). (**C**) Effect of peptide concentration on antibody blocking and RBD-ACE2 interaction. The assay was performed with three different concentrations (0.1, 1, 5 nmole/100 uL reaction) of peptide and the study with serum from vaccinated monkey number 4 is shown as a representative. (**D**) Percent change in RBD-ACE2 interaction in the presence of peptide. Percent change was calculated based on the formula described in the materials and methods.

Additionally, peptides of the three immunodominant epitopes were added to competitively inhibit antibody binding to the RBD protein (Fig. 3B, right panel). When tested with sera from monkeys number 1 and 2, we observed a trend that the addition of peptides CoV2_S-10, CoV2_S-11 and CoV2_S-11.2 increased RBD-ACE2 binding (Fig. 3B, right panel). In contrast, the addition of the peptide CoV2_S-13 resulted in a decrease of RBD-ACE2 binding. However, serum from vaccinated monkey 4, which has a lower antibody response against those three epitopes (Fig. 3B, left panel), exhibited a clearer effect of antibody blocking (Fig. 3B-C). Addition of the peptides CoV2_S-10, CoV2_S-11 and CoV2_S-11.2 resulted in a significant increase of RBD-ACE2 interaction. For epitope CoV2_S-13, although the level of antibody against this epitope was highest in monkey serum 4, the addition of the peptide CoV2_S-13 showed the lowest increment of RBD-ACE2 binding, compared to other peptides.

In addition, we also investigated whether different peptide concentrations affect antibody blocking. Our results showed that increased peptide concentrations, moderately reduced RBD-ACE2 binding (Fig. 3C). The percent increase in the RBD-ACE2 interaction was calculated (Fig. 3D). Antibody blocking with the peptides CoV2_S-10, CoV2_S-11 and CoV2_S-11.2 showed an increase in RBD-ACE2 binding in all tested sera. The largest increase across the experiment was 21%, as observed when using sera from vaccinated monkey number 4 and blocking with peptide CoV2_S-10. Taken together, this experiment demonstrates that antibodies recognizing epitopes CoV2_S-10, CoV2_S-11 and CoV2_S-11.2 are capable of inhibiting the interaction between SARS-CoV-2 RBD and ACE2, while antibody targeting epitope CoV2_S-13 tends to have only a low effect on RBD-ACE2 interaction.

### Development of an immunoinformatics method for identification of linear B cell epitope

**Figure 4:**
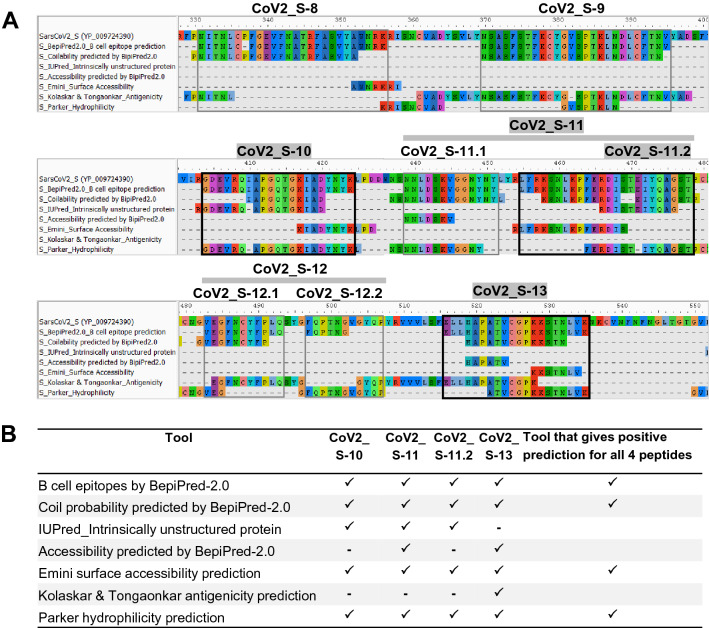
Characterization of the immunodominant epitopes using multiple immunoinformatics tools. In addition to linear B cell epitope prediction with BepiPred-2.0, the entire sequence of the SARS-CoV-2 RBD was predicted for coil structure, accessibility, antigenicity and hydrophilicity using the prediction tools as indicated. (**A**) Alignment of the sequences obtained from each prediction tool. The immunodominant epitopes are indicated (thick box). (**B**) Determination of common characteristics of the three immunodominant epitopes.

As several parameters such as coil structure, surface exposure/accessibility, hydrophilicity, flexibility, and antigenic propensity have been suggested to be correlated with the location of B cell epitopes^28,29^, we further predicted characteristics of the immunodominant epitopes using multiple immunoinformatics tools (see materials and methods). To search for common characteristics among the three immunodominant epitopes (four peptides tested), the sequences predicted by each tool were aligned to the sequence of epitopes predicted by BepiPred-2.0 (Fig. 4A). As shown in Fig. 4B, all four peptides exhibited the following common characteristics: (i) predicted as an epitope by BepiPred-2.0, (ii) predicted to be in coil structure by BepiPred-2.0, (iii) predicted to be hydrophilic by the method of Parker, and (iv) predicted to be exposed on the surface by the method of Emini. Based on this observation, we then developed a new and simple immunoinformatics-based method for identifying linear B cell epitopes, which is designated as method A. In this method, linear B cell epitope is identified based on the peptide predicted by BepiPred-2.0 that has at least four residues overlapping with the peptides predicted by the other 3 tools.

### Prediction of linear B cell epitopes in the SARS-CoV-2 structural proteins

**Figure 5:**
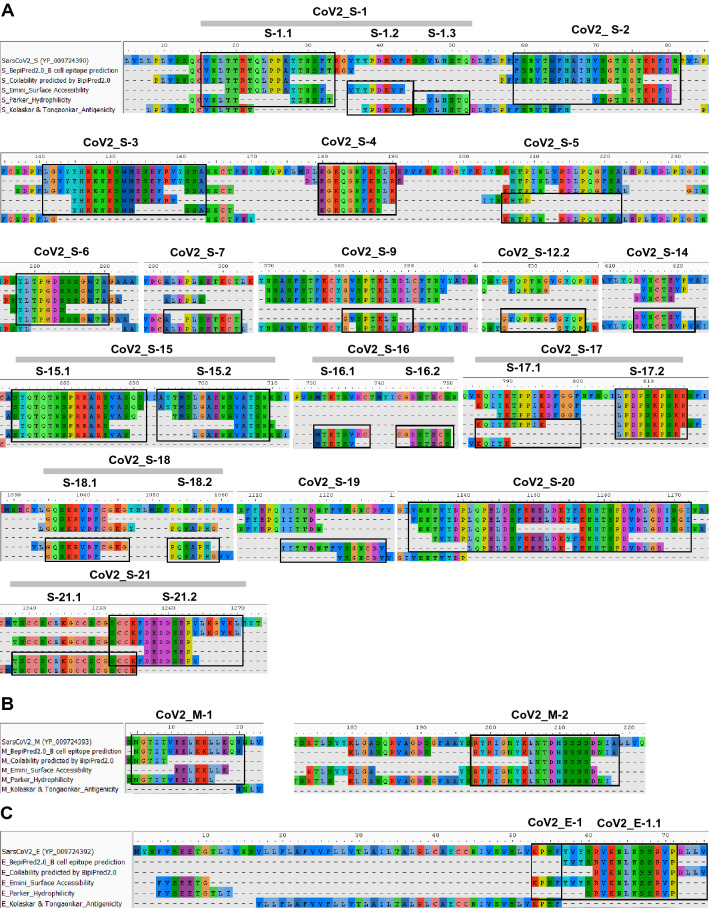
Prediction of linear B cell epitope in the S, M and E proteins using immunoinformatics approach. Amino acid sequences of the SARS-CoV-2 S (**A**), M (**B**) and E (**C**) proteins were predicted with the tools indicated and then aligned. Linear B cell epitopes were identified based on the bioinformatics methods A, B and C. Selected sequences are indicated (in boxes) with designated epitope names.

We further predicted potential linear B cell epitopes in the SARS-CoV-2 structural proteins using the immunoinformatics methods. Along with method A developed in this study, we employed other 2 methods, designated method B and C, the methods we created in our previous work to identify B cell epitopes of the proteins from porcine epidemic diarrhea virus, a member of alphacoronavirus (manuscript submitted). For method B, linear B cell epitope is identified based on the peptides with antigenic and hydrophilic determinants predicted by the methods of Kolaskar & Tongaonkar and Parker, respectively (Fig. 5). For method C, the peptide with antigenic and surface-exposed determinants obtained from predictions using the methods of Kolaskar & Tongaonkar and Emini, respectively, are predicted as B cell epitopes.

All three immunoinformatics methods were employed to predict B cells epitopes in the SARS-CoV-2 structural proteins S, M and E. Predicted linear B cell epitopes are shown in Fig. 5A-C. The sequences and further characteristics of the predicted epitopes are shown in Table 1. In the RBD, besides the epitopes identified by the method A shown in Fig. 4, prediction with method B gave rise to 2 additional epitopes, CoV2_S-9 and CoV2-S-12.2, (Fig. 5A). Two predicted epitopes, CoV2_M-1 and CoV2_M-2, were obtained from analysis of the M protein (Fig. 5B). Examination of the E protein revealed one region at the C-terminus with potential as a B cell epitope (Fig. 5C).

### Validation of the predicted B cell epitopes with COVID-19 convalescent sera

**Figure 6:**
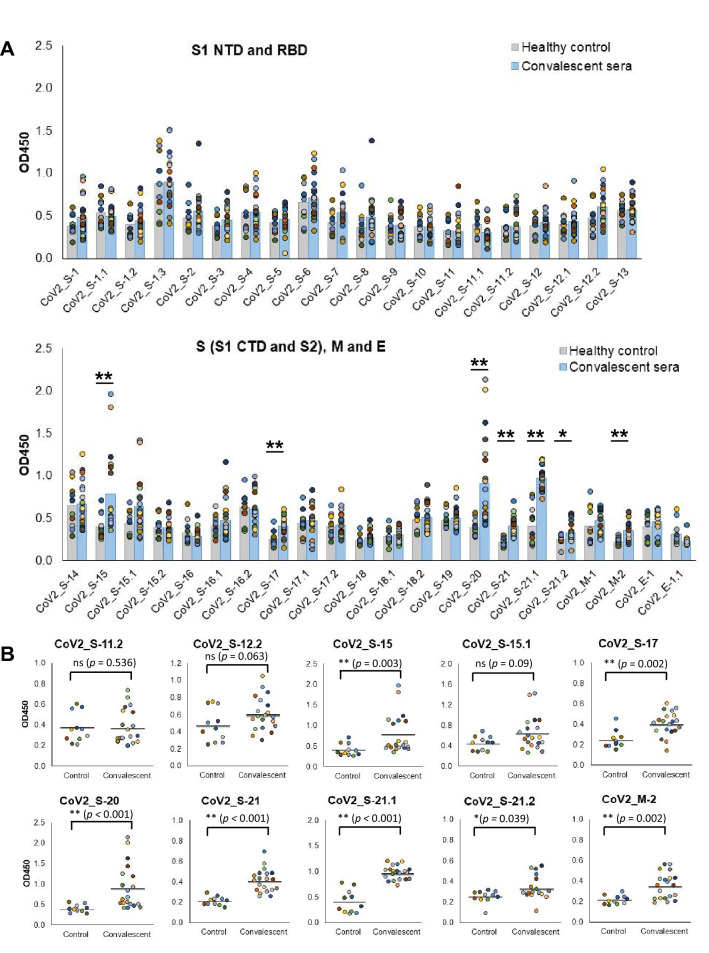
Antibody responses against the predicted B cell epitopes in COVID-19 convalescent sera. Peptides with the sequence corresponding to the predicted linear B cell epitopes were tested with 20 convalescent serum samples and 11 serum samples from healthy individuals using ELISA. (**A**) Antibody responses against each predicted B cell epitope on the S, M and E proteins. Antibody responses in healthy control sera and convalescent sera were compared and statically tested using non-parametric Mann–Whitney test. “*” and “**” represent a significant difference between the two groups with *p*-value < 0.05 and < 0.001, respectively. (**B**) Antibody responses against epitopes CoV2_S-11.2 and CoV2_S-12.2 located within the RBD and immunodominant epitopes. Antibody response in individual serum and the result of statistical analysis are shown. ns: not significant (*p* > 0.05).

Predicted B cell epitopes were next validated with COVID-19 convalescent sera. ELISA was performed by testing synthetic peptides with 20 COVID-19 convalescent sera with confirmed neutralizing activity and 11 serum samples from individuals unexposed to SARS-CoV-2 (healthy control). Statistical analysis revealed that antibody responses against epitopes CoV2_S-15, CoV2_S-17, CoV2_S-20, CoV2_S-21, CoV2_S-21.1, CoV2_S-21.2 and CoV2_M-2 in convalescent sera are significantly higher than those in healthy control sera (*p* < 0.05) (Fig. 6A,B). Thus, these epitopes are considered immunodominant. Among these 7 immunodominant epitopes, CoV2_S-15 and CoV2_S-20 elicit potent antibody response as seen with high OD450 in several sera. On the other hand, the majority of SARS-CoV-2-infected patients tend to respond to epitopes CoV2_S-17 and CoV2_S-21, although the OD values in the convalescent group and control group were not markedly different.

We went on to determine the number of convalescent sera patients with antibody responses against each individual epitope, as defined by OD450 of convalescent sera being greater than OD450 of control group mean. The number of epitope-responding sera of each epitope is summarized in Table 1. Almost all epitopes reacted with at least one convalescent serum, suggesting their capability to elicit an antibody response in SARS-CoV-2-infected patients. In the RBD, epitope CoV2_S-12.2 was most potent in eliciting antibody response in individuals naturally infected with SARS-CoV-2, although the responses in convalescent and control sera were not significantly different (*p* = 0.08). On the other hand, epitope CoV2_S-11.2 showed positive reactivity with 6 convalescent sera.

**Figure 7:**
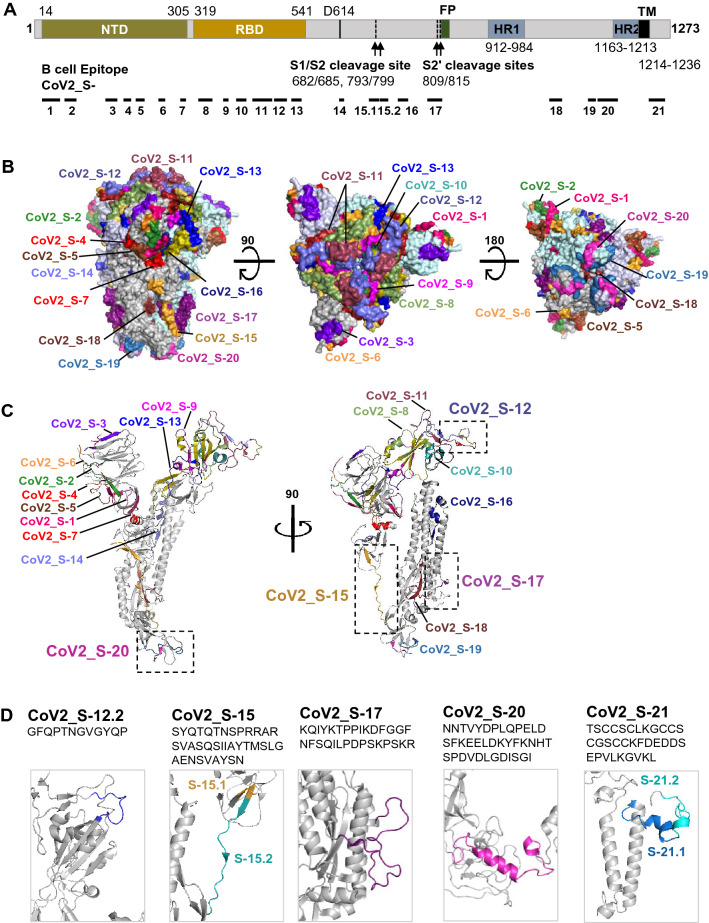
Schematic representation of the SARS-CoV-2 S protein and locations of linear B cell epitope on the S protein. (**A**) Schematic location of the B cell epitopes on the S protein. (**B**) Surface representation of the B cell epitopes on the SARS-CoV-2 S trimer. (**C**) Location of the B cell epitopes in the SARS-CoV-2 S monomer. (**D**) Secondary structure of the immunodominant B cell epitopes. Closed conformation of the S protein (PDB 6ZB5) is used for epitope labeling. RBD of each subunit and B cell epitopes are illustrated in different colors. Note that 3-D structure of epitopes CoV2_S-20 and CoV2_S-21 were predicted and computationally modeled.

Locations of the epitopes in the S protein are shown in Fig. 7A. Surface depiction of the B cell epitopes on the trimeric S protein demonstrated that most of the B cell epitopes we identified are exposed on the surface of the S protein (Fig. 7B). In addition, locations and secondary structures of each epitope were also depicted on the monomeric S protein (Fig. 7C and Supplementary Fig. 2 and 3). Close-ups of the immunodominant epitopes and epitope CoV2_S-12.2 suggest the presence of coil structure in the epitopes (Fig. 7D).

### Localization of the amino acid changes in the B cell epitopes in variants of concern

Amino acid substitutions and deletions on the S protein of four variants of concern B.1.1.7 (Alpha), B.1.351 (Beta), P.1 (Gamma), and B.1.617.2 (Delta) were localized and mapped to the B cell epitopes on the 3-D structure of wild type (Wuhan) SARS-CoV-2 S protein (Fig. 8). The occurrence of amino acid changes in all four variants is mainly found in the S1 domain, which includes RBD, NTD and CTD. In the B.1.1.7 variant, amino acid substitutions are found in the epitopes CoV2_S-12.2, -14, -15.1 and -19, while deleted residues are parts of epitopes CoV2_S-2, and CoV2_S-3. In the B.1.351 variant, amino acid substitutions are found in epitopes CoV2_S-1, -2, -5, -10, -12.1, 12.2, -14, and -15.2. The new emerging variant of concern, B.1.617.2 has amino acid changes in multiple important epitopes. Epitope CoV2_S-3 in the B.1.617.2, variant is potentially affected by 2 deletions and 2 amino acids substitution, while epitopes CoV2_S-1.1, 10, 11, 11.2, 14, 15.1 are possibly affected by amino acid substitutions. The P.1 variant has fewer amino acid changes in the epitope regions compared to the other 3 variants, by which amino acid residues in the epitopes CoV2_S-1, -4, -10, -12, and -14 are substituted with other amino acids.

**﻿Figure 8:**
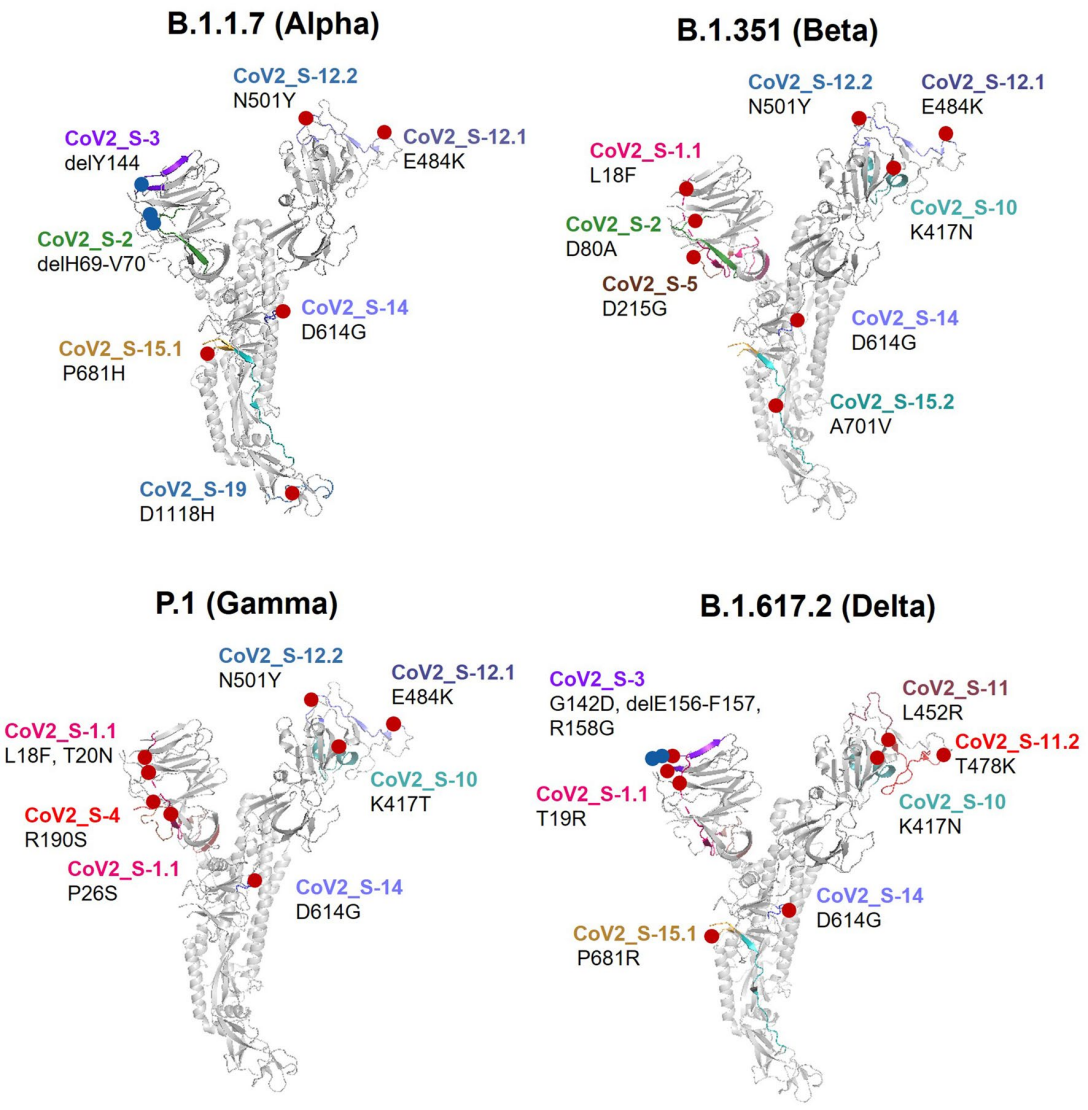
B cell epitopes in the Wuhan SARS-CoV-2 S protein with amino acid changes found in variants of concern. The structural cartoons depict amino acid substitutions and deletions in the S protein of four variants of concern (B.1.1.7 (Alpha), B.1.351 (Beta), P.1 (Gamma) and B.1.617.2 (Delta)) relative to location of B cell epitopes in the Wuhan SARS-CoV-2 S protein. Position of the amino acids is based on the Wuhan SARS-CoV-2 sequence. Locations of the amino acid substitutions and deletions on the structural cartoon are indicated with red and blue circles, respectively.

Lastly, we employed the immunoinformatics methods we developed to explore the B cell epitope profile in the four variants of concern (Supplementary Fig. 4). As a result of amino acid changes, some epitopes such as CoV2_S-4 in the NTD and CoV2_S-10 in the RBD could no longer be predicted as epitopes in the P.1 and B.1.617.2 variants. Epitopes CoV2_S-11 and CoV2_S-12 in different variants were predicted to be different regarding length and position.

Altogether, our analyses demonstrate that amino acid deletions and substitutions in the SARS-CoV-2 variants of concern are found in many epitopes in the S protein and may contribute to immune escape of these new variants.

## Discussion

Although SARS-CoV-2 S protein is considered the best candidate for vaccine development, it is a large and complex glycoprotein, thereby limiting its manufacturing yield and requiring a suitable host for protein production. RBD, however, which is a small domain (aa 319–541) that plays an important role in the primary interaction with the host cell receptor ACE2, is a more attractive alternative for vaccine development. Recombinant RBD subunit vaccines have been shown to stimulate immune responses in mice, rabbits and non-human primates and also protected *Macaca mulatta* from SARS-CoV-2 pseudovirus and live SARS-CoV-2^30^. The company Baiya Phytopharm has developed a plant-produced RBD subunit vaccine, which exhibited an ability to induce anti-RBD antibodies resulting in SARS-CoV-2 neutralization in mouse and non-human primate *M. fascicularis* models^26^. Due to its central function in viral biology, RBD has become a focus in host cell entry, immune induction, and immune escape studies by new variants. Thus, profiling of B cell epitopes in the RBD is of great importance and benefit to various aspects of SARS-CoV-2 studies. However, it has been consistently reported that in COVID-19 patients, epitopes in the RBD are less dominant compared to the S2 domain^16,31,32^. Despite extensive studies on antibody response against the RBD, B cell epitope profile in this domain is still unclear.

Serological assays with sera from cynomolgus macaques vaccinated with plant-produced RBD subunit revealed three immunodominant linear B cell epitopes located in the RBD of the SARS-CoV-2 S protein. Interestingly, in COVID-19 patients, these three epitopes are recognized only as subdominant ones, while other peptides in the S1 CTD and S2 have been demonstrated as immunodominant^16,32–34^. These results suggest that natural infection and vaccination could result in different patterns and levels of antibody responses. While natural infection can give rise to antibody responses against the whole viral proteome, RBD-based vaccines contain only a few epitopes and has no competitive interference from epitopes in other proteins. Thus, utilization of the RBD as an immunogen represent a better strategy to enhance antibody response against those subdominant epitopes in the RBD.

To address neutralizing potential of the antibodies targeting these three RBD immunodominant epitopes, we performed competitive inhibition of RBD-ACE2 binding. The results showed that antibodies targeting epitopes CoV2_S-10 and CoV2_S-11 were capable of inhibiting RBD-ACE2 interaction, thus suggesting their neutralizing potency. Interestingly, the opposite effect on an interaction between RBD and ACE2 was observed for CoV2_S-13 epitope. This is not surprising because residues in the CoV2_S-13 epitope are not directly responsible for interacting with the host cell receptor ACE2^35,36^. Decreased RBD-ACE2 interaction in the presence of peptide CoV2_S-13 may be due to the replacement of antibodies targeting this epitope with antibodies targeting other epitopes such as CoV2_S-10 and CoV2_S-11, which can inhibit RBD-ACE2 binding. As a result, binding of the RBD to ACE2 was further inhibited (this assumes that only one antibody can bind to the RBD protein at a time). However, antibodies targeting the CoV2_S-13 epitope may be a hindrance at other processes of SARS-CoV-2 infection such as conformational changes and membrane fusion^37^, which occur after the binding of RBD to ACE2 receptor. As sera from these vaccinated monkeys were demonstrated to neutralize SARS-CoV-2^26^, it is possible that neutralizing activity in these sera mainly results from antibodies against the three immunodominant epitopes we identified in this study. However, there may be other neutralizing epitopes in the RBD, particularly in the form of conformational epitopes.

Although competitive inhibition of RBD-ACE2 binding can hint towards neutralizing potency of antibodies recognizing RBD epitopes, it is still necessary to perform a SARS-CoV-2 neutralization-inhibition assay to confirm protective immune response. However, neutralizing activity of antibodies against some epitopes in the RBD have been reported and confirmed. It has been shown that the peptide 404-GDEVRQIAPGQTGKIADYNYKL-425, which overlaps entirely with our epitope CoV2_S-10, could elicit neutralizing antibody responses in mice^38^. Moreover, monoclonal antibody B38^14^, which can neutralize SARS-CoV-2, showed interaction with multiple residues in the RBD including the residues in the CoV2_S-11 epitope. Moreover, murine antibodies induced by peptides 406–420 (EVRQIAPGQTGKIAD), 439–454 (NNLDSKVGGNYNYLYR) and 455–469 (LFRKSNLKPFERDIS), which correspond to our epitopes CoV2_S-10, CoV2_S-11.1 and CoV2_S-11.2, respectively, inhibited SARS-CoV-2 pseudovirus infection^31^.

Testing the synthetic peptides corresponding to the predicted epitopes in the S and M proteins with convalescent sera allowed us to determine their immunodominant properties. In agreement with other studies, our results showed that linear B cell epitopes in the RBD are less dominant compared to those within the S2 domain^16,34^. Here, we reported six immunodominant epitopes (CoV2_S-15, -17, -20, -21, -21.1 and -21.2) located in four regions of the S protein and one immunodominant epitope located at the C-terminus of the M protein. Importantly, these immunodominant epitopes are in the functionally important regions of the S1 CTD and S2 domain. Epitope CoV2_S-15 (673-SYQTQTNSPRRARSVASQSIIAYTMSLGAENSVAYSN-709) is located at the S1/S2 cleavage site, where the sequence RRAR can be recognized and cleaved by furin protease, resulting in separation of S1 and S2 domains during virus assembly^39,40^. In addition, this epitope region also contains cleavage site 2 IAYTMSL, which is recognized and cleaved by Cathepsin L in the endosome and this process is also essential in S protein priming during cell entry^41^. Thus, antibodies targeting epitope CoV2_S-15 could possibly block the cleavage processing of the S protein during cell entry of the virus. Epitope CoV2_S-17 is found in the S2’ cleavage site (residues 809–815), the target for cleavage by proteases including TMPRSS2, a serine protease, for S protein prime^42,43^. Consequently, antibodies binding to this region may result in inhibition of the S2’ cleavage, a crucial process during viral-cell entry. The epitope 809-PSKPSKRSFIEDLLFNKV-826, which overlaps with epitope CoV2_S-17, has been demonstrated as a neutralizing epitope^33^. Epitope CoV2_S-20 is located at the upstream region of the HR2, a domain involving in membrane fusion of SARS-CoV-2 during virus entry^44,45^. Antibodies against this epitope may lead to inhibition of the membrane fusion and virus infection. Epitopes that overlap with CoV2_S-20 have been previously characterized as immunodominant as well as neutralizing epitopes^16,34^. Another immunodominant epitope CoV2_S-21, which is located at the cytoplasmic domain of the S protein, has also been identified as one of the immunodominant epitopes in other studies^16,34^. Although this epitope has not been clarified to elicit neutralizing antibodies, research into an alphacoronavirus suggests its potential. B cell epitopes located at the cytoplasmic domain of the S protein of the porcine epidemic diarrhea virus (PEDV), a member of alphacoronaviruses, is shown to be a target for recognition by the neutralizing antibody 2C10^46^. It remains possible that the epitope CoV2_S-21, which is in the same location as epitope 2C10, can elicit antibodies with neutralizing activity. For the immunodominant epitope in the M protein, further studies are needed to address whether the antibodies elicited by this epitope confer neutralizing activity. Based on these findings, the immunodominant epitopes CoV2_S-15, -17, -20 and -21 may represent valuable candidates for both vaccine development and assessment of the antibody response in COVID-19 patients and vaccine recipients.

Here, we demonstrated that our immunoinformatics method is a valuable tool for predicting linear B cell epitopes both in wild type virus and new emerging strains. Mapping amino acid changes in the S protein of variants of concern to our B cell epitopes revealed that sequences within the S1 NTD, RBD and S1/S2 cleavage site are the main targets for amino acid substitutions and deletions. Some of these B cell epitopes with amino acid changes have been well characterized as neutralizing epitopes. For instance, epitopes CoV2_S-10 and -12 are located in the RBD. Additionally, the CoV2_S-3 epitope is a part of the epitope recognized by monoclonal neutralizing antibody 4A8^19^. Amino acid substitutions P681H in B.1.1.7 and P681R in B.1.617.2 are located in the CoV2_S-15.1 epitope that contains the sequence RRAR for S1/S2 furin cleavage, while A701V in the B.1.351 variant is located in the CoV2_S-15.2 epitope, which contains Cathepsin L cleavage site^41^. These findings may help explain why these new variants are more resistant to antibodies induced by either natural infection or by vaccines based on wild type SARS-CoV-2 as reported in several studies^7,8,10^.

Taken together, we identified three linear B cell epitopes in the RBD of the SARS-CoV-2 S protein by coupling the immunoinformatics approach with the immunoassay testing sera from macaques vaccinated with plant-produced RBD. This combined method enabled the identification of six immunodominant epitopes in the S1/S2 cleavage site and S2 domain recognized by antibodies in the COVID-19 convalescent sera. The immunoinformatics method described here could be a useful tool for identification of antibody epitopes in new virus variants and also other target proteins. Linear B cell epitopes discovered in this study may find future applications both in designing a new candidate SARS-CoV-2 vaccine focusing on antibody induction and in development of peptide-based immunoassays.

## Materials and methods

### COVID-19 convalescent sera

Serum samples used in this study had been previously used in the project “measurement of neutralizing antibody in convalescent plasma donors for COVID-19 treatment”, which was approved by the Siriraj Institutional Review Board under COA number Si 483/2020. Informed consent was waived by the Institutional Review Board. All methods were carried out in accordance with international guidelines for human research protection, the declaration of Helsinki. Twenty serum samples collected from recovered Thai COVID-19 patients with positive neutralizing antibody test were chosen and used in this study while 11 control serum samples were prepared from healthy individuals with negative test for COVID-19. All sera were heat-inactivated at 56 °C for 30 min before used.

### Vaccine and monkey sera

Recombinant SARS-CoV-2 RBD subunit produced from plant is a SARS-CoV-2 vaccine candidate developed by Baiya Phytopharm Co., Ltd. Preclinical evaluation of this vaccine was conducted in mice and cynomolgus macaques^26^. Monkey sera used in this study were provided by Baiya Phytopharm Co., Ltd, under the project “Immunogenicity study of low dose of plant-produced recombinant SARS-CoV-2 RBD subunit vaccine in cynomolgus macaques (*Macaca fascicularis*)”. Serum samples were collected from 8 monkeys vaccinated with plant-produced RBD subunit vaccine adjuvanted with Alum and 5 monkeys in the control group receiving PBS adjuvanted with Alum^26^. The study was carried out in compliance with ‘The Animals in research: reporting in vivo experiments (ARRIVE)’ Guidelines. All procedures in non-human primate study were conducted in accordance with relevant guidelines and regulations, which were reviewed and approved by the NPRCT (National Primate Research Center of Thailand), Chulalongkorn University (NPRCT-CU) Animal Care and Use Committee (Protocol review No. 207512). The NPRCT facility has been AAALAC International Accredited (1752).

### B cell epitope prediction using BepiPred-2.0

The amino acid sequence of the S protein (accession number YP_009724390), M protein (accession number YP_009724393) and E protein (YP_009724392) of SARS-CoV-2 were retrieved from National Center for Biotechnology Information (NCBI). The sequences and mutations of the S protein of the variants of concern, B.1.1.7, B.1.351, P.1 and B.1.617.2 were obtained from Center for Disease Control and Prevention (CDC)^6^ and preliminary finding reported in the virology.org^47^.

B cell epitopes were predicted using BepiPred-2.0, (http://www.cbs.dtu.dk/services/BepiPred/). BepiPred-2.0 is an immunoinformatics tool used for predicting continuous/linear B cell/antibody epitope from protein sequences^48^. In this work, we used the cutoff of 0.5 and the epitope was defined based on the predicted sequence that contains at least 6 contiguous residues. It is noteworthy that prediction using BepiPred-2.0 tool through its original server (http://www.cbs.dtu.dk/services/BepiPred/) and the server provided by IEDB (http://tools.iedb.org/bcell/) could give a slight difference in the prediction result.

### Immunoinformatics prediction with multiple tools

Other characteristics including coil structure, surface exposure/accessibility, antigenicity and hydrophilicity were predicted with multiple immunoinformatics tools. Coil structure was predicted with two tools, BepiPred-2.0 and IUPred. BepiPred 2.0 provides coil probability score of each amino acid residue, while IUPred (https://iupred.elte.hu/) predicts intrinsically unstructured proteins, which infers coil structure^49^. In the prediction with the IUPred tool, the residues of the S protein predicted with disorder prediction score higher than 0.2 were considered unstructured. Surface accessibility was predicted with two tools, BepiPred-2.0 providing the probability of exposed residues and the method of Emini providing probability for being found on the surface^50^. Antigenicity propensity was predicted by the method of Kolaskar & Tongaonkar, a semi-empirical method^51^. Hydrophilicity was predicted by the method of Parker^52^. Notably, the method of Emini, Kolaskar & Tongaonkar, and Parker are provided by IEDB (http://tools.iedb.org/bcell/) and the default cutoff thresholds provided by the programs were used to select the sequences. In all prediction methods, only the predicted peptides with at least 6 contiguous residues were selected and used for further analysis. All peptides obtained from predictions with different tools were aligned using AliView program^53^ and a method for selection of potential B cell epitopes were then created.

### Peptide and protein preparation

Peptides with the sequences corresponding to the predicted epitopes were chemically synthesized (Mimotopes, Australia). Synthetic peptides were dissolved in sterile distilled water containing 0.1% acetic acid to the concentration of 3 nmol/μL (3 mM) and stored at -20 °C until used. The RBD subunit used in the ELISA assay was produced in the mammalian HEK 293 cell growing in DMEM supplemented with 10% fetal bovine serum (HyClone) and 1% penicillin/streptomycin antibiotics (Gibco). The cells were transfected with the plasmid pVAX1 harboring a gene encoding SARS-CoV-2 RBD subunit. As the protein was designed to be extracellularly secreted, the culture medium was harvested. The presence of the RBD subunit in the medium was confirmed by Western blot. Protein concentration in the harvested medium was measured using Bradford assay (Bio-Rad).

### Enzyme-linked immunosorbent assay (ELISA)

ELISA microplates (Greiner bio-one) were coated with 1 nmole of synthetic peptides or 20 μg of the RBD protein (in medium) diluted in 50 μL PBS. Medium without RBD protein was used as a negative control. Following an overnight incubation at 4 °C, plates were washed 3 times with PBS containing 0.05% Tween 20 (PBST) and blocked with 100 μL PBST containing 5% FBS (5% FBS/PBST) for 1 h at room temperature (RT). Sera were diluted 1:100 in PBST containing 1% FBS (1% FBS/PBST) and added to the plates (100 μL/well) (in duplicate for monkey sera and one well for human sera) and incubated at RT for 2 h. After a 3-time wash, goat anti-monkey IgG HRP antibody (Abcam) diluted 1:10,000 in 1% FBS/PBST or rabbit anti-human IgG HRP antibody (Abcam) diluted 1:80,000 was added to the well (100 μL/well) and the plates were incubated at RT for 90 min. After washing, TMB substrate (BioLegend) was added (70 μL/well) to develop color. Following a 30-min incubation, the reaction was stopped by adding 30 μL 2 N sulfuric acid (H_2_SO_4_). Optical Density at the wavelength of 450 nm (OD450) was measured (MULTISKAN FC, Thermo scientific).

### Competitive inhibition of ACE2-RBD interaction

ELISA microplates (Greiner bio-one) were coated with 4 μg/mL (50 μL/well) of recombinant human ACE2 (ab273687, Abcam). Following an overnight incubation at 4 °C, plate was washed 3 times with PBST and blocked with 100 μL 5% FBS/PBST for 1 h at RT. After washing, RBD protein (2.5 μg crude protein in DMEM) prepared in a final volume of 100 μL in 3 different conditions (i) RBD alone, (ii) RBD mixed with monkey serum (diluted 1:500), and (iii) RBD mixed with monkey serum and peptide, was added into each well and incubated for 1 h. The experiment was conducted in triplicate. After a 3-time wash, mouse anti-V5 antibody (Invitrogen) diluted 1:2,000 in 1% FBS/PBS was added and the plate was incubated for 1 h at RT. After washing, goat-anti mouse IgG-HRP (Abcam) diluted 1:100,000 was added to the well (100 μL/well) and incubated for one hour. The following steps followed ELISA method as described above. Percent change was calculated based on the formula: 100 x (OD of RBD with serum and peptide - OD of RBD with serum)/(OD of RBD – OD of RBD with serum).

### Localization of the B cell epitopes on the SARS CoV-2 S protein

Surface presentation of the B cell epitopes on the SARS CoV-2 S protein was depicted on the 3-D structure of trimeric SARS CoV-2 S protein in the closed conformation (PDB 6ZB5^27^) obtained from https://www.rcsb.org/. Epitope labeling was performed using PyMOL 2.3.4 program. Additionally, locations of the epitopes were depicted on the monomeric SARS CoV-2 S protein in both closed (PDB 6ZB5^27^) and open (PDB 6VYB^54^) conformations.

### Statistical analysis

Statistical analyses of each experiment are reported in the results and figure legends. The statistical significance of two different groups was analyzed using SPSS 22 for Windows software (SPSS, USA). Parametric students’ t-test was used to analyze the results of ELISA testing monkey sera, while non-parametric Mann–Whitney test was used to analyze ELISA result of human sera. *p* < 0.05 was considered statistically significant.

## Supplementary Information

Supplementary Information.
