# Supplementary material for: Immunodominant linear B cell epitopes in the spike and membrane proteins of SARS-CoV-2 identified by immunoinformatics prediction and immunoassay
Source: Sci Rep. 2021 Oct 14;11:20383. doi: 10.1038/s41598-021-99642-w (PMC8516869; doi:10.1038/s41598-021-99642-w)
Supplement: Supplementary file 1 — Supplementary Information. [file 41598_2021_99642_MOESM1_ESM.docx]

**Supplementary information**


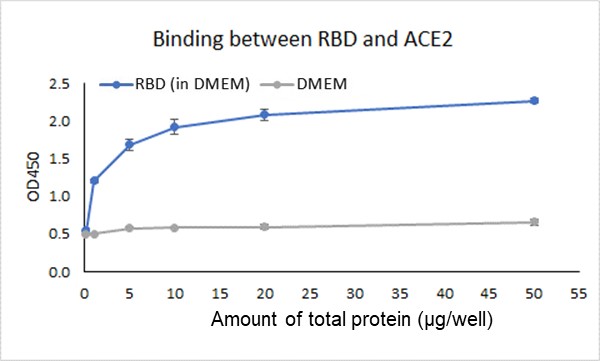


**Supplementary Figure 1.** Interaction between RBD and human ACE2. Different concentrations of the RBD (crude protein in DMEM medium) ranging from 0.1 to 50 µg in 100 µl/well were added to the ELISA plate coated with recombinant human ACE2. DMEM collected from from HEK 293 culture was used as a negative control. The bound RBD protein was then detected with mouse anti-V5 antibody, followed by anti-mouse IgG HRP. The optical density at the wavelength of 450 nm (OD450) was next measured.


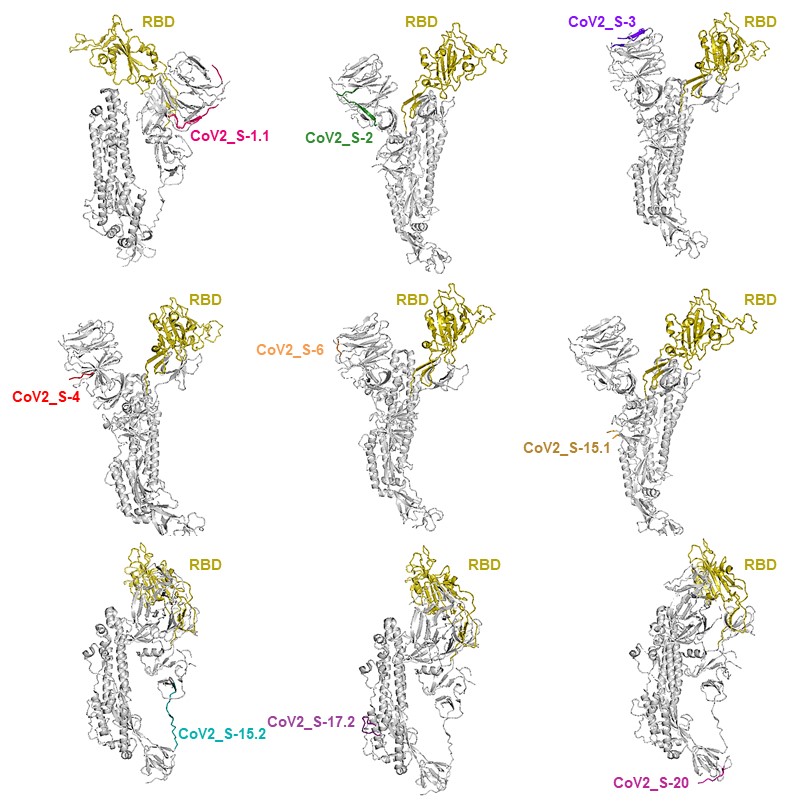


**Supplementary Figure 2.** Locations and of the epitope identified by the immunoinformatics method A. Location and of the epitope of each epitope are depicted on the SARS-CoV-2 monomeric S protein using PyMOL 2.3.4 program. Notably, the epitope CoV2_S-21 is not shown because the cytosolic C-terminal domain is excluded from the S protein used in 3-D structure structure.


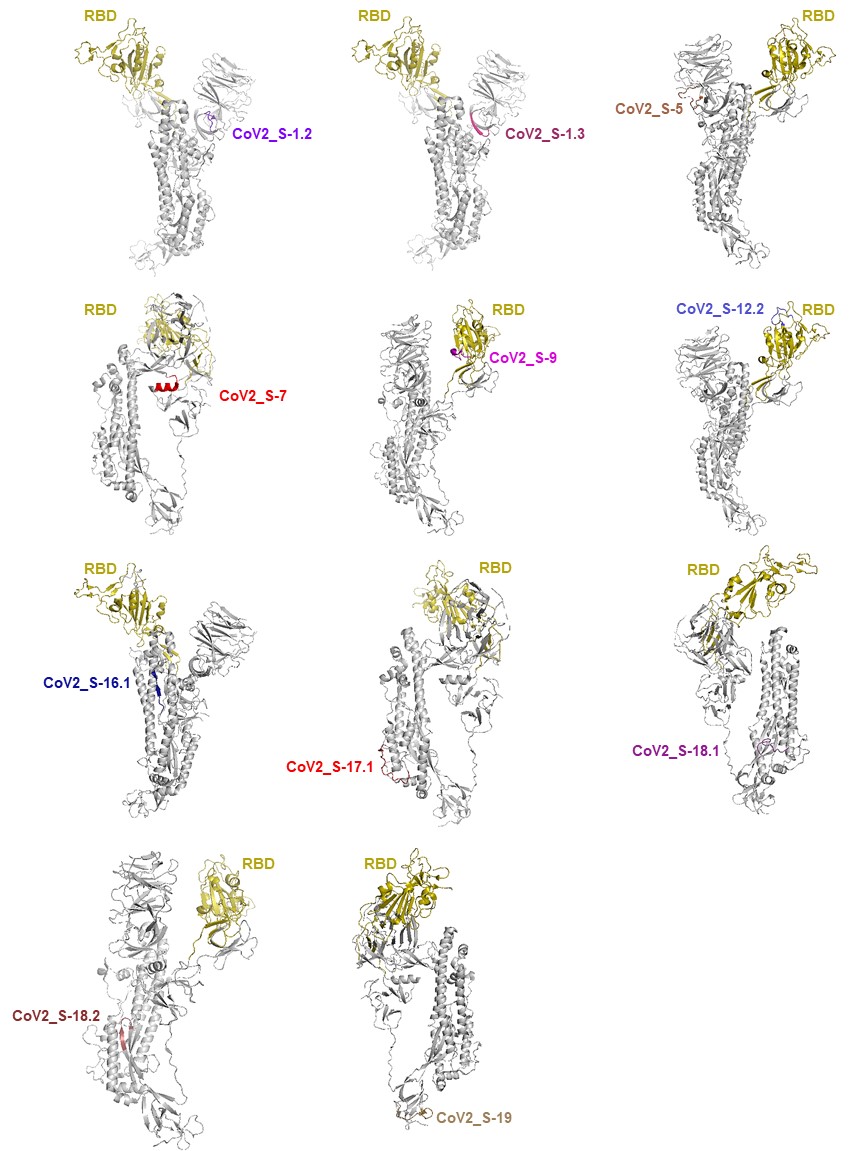


**Supplementary Figure 3.** Locations and structures of the epitopes identified by the immunoinformatics method B and C. Location and structure of each epitope are indicated on the SARS-CoV-2 monomeric S protein using PyMOL 2.3.4 program.


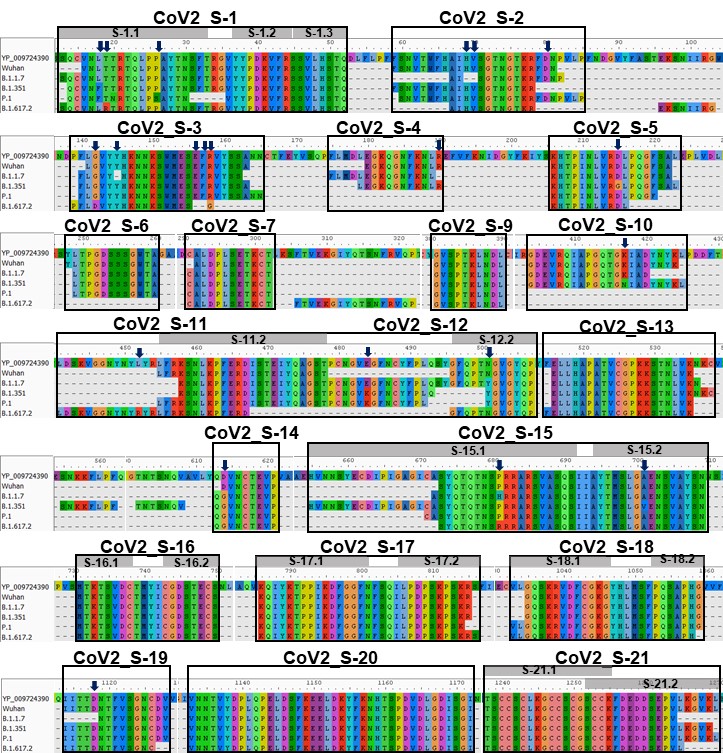


**Figure 4. Prediction of B cell epitopes in the S protein of four SARS-CoV-2 variants of concerns .** Based on our immunoinformatics approach, B cell epitopes were identified from the S protein of four SARS-CoV-2 variants of concern: B.1.1.7, B.1.351, P.1, and B.1.617.2. Resulting epitopes from each variant are aligned. Arrows indicate the positions with amino acid changes (either amino acid substitution or deletion) found in the variants of concern. Epitopes are boxed and named.
